# Changes in water color shift competition between phytoplankton species with contrasting light‐harvesting strategies

**DOI:** 10.1002/ecy.2951

**Published:** 2020-02-03

**Authors:** Veerle M. Luimstra, Jolanda M. H. Verspagen, Tianshuo Xu, J. Merijn Schuurmans, Jef Huisman

**Affiliations:** ^1^ Department of Freshwater and Marine Ecology Institute for Biodiversity and Ecosystem Dynamics University of Amsterdam PO Box 94240 Amsterdam 1090 GE The Netherlands; ^2^ Wetsus European Centre of Excellence for Sustainable Water Technology Oostergoweg 9 Leeuwarden 8911 MA The Netherlands

**Keywords:** blue light, coexistence, cyanobacteria, green algae, lake brownification, light spectrum, light‐harvesting antennae, niche differentiation, photosynthesis, *Prochlorococcus*, resource competition, *Synechococcus*

## Abstract

The color of many lakes and seas is changing, which is likely to affect the species composition of freshwater and marine phytoplankton communities. For example, cyanobacteria with phycobilisomes as light‐harvesting antennae can effectively utilize green or orange‐red light. However, recent studies show that they use blue light much less efficiently than phytoplankton species with chlorophyll‐based light‐harvesting complexes, even though both phytoplankton groups may absorb blue light to a similar extent. Can we advance ecological theory to predict how these differences in light‐harvesting strategy affect competition between phytoplankton species? Here, we develop a new resource competition model in which the absorption and utilization efficiency of different colors of light are varied independently. The model was parameterized using monoculture experiments with a freshwater cyanobacterium and green alga, as representatives of phytoplankton with phycobilisome‐based vs. chlorophyll‐based light‐harvesting antennae. The parameterized model was subsequently tested in a series of competition experiments. In agreement with the model predictions, the green alga won the competition in blue light whereas the cyanobacterium won in red light, irrespective of the initial relative abundances of the species. These results are in line with observed changes in phytoplankton community structure in response to lake brownification. Similarly, in marine waters, the model predicts dominance of *Prochlorococcus* with chlorophyll‐based light‐harvesting complexes in blue light but dominance of *Synechococcus* with phycobilisomes in green light, with a broad range of coexistence in between. These predictions agree well with the known biogeographical distributions of these two highly abundant marine taxa. Our results offer a novel trait‐based approach to understand and predict competition between phytoplankton species with different photosynthetic pigments and light‐harvesting strategies.

## Introduction

Phytoplankton provide the base of the food web in freshwater and marine ecosystems (Sterner and Elser 2002, Falkowski 2012), and their photosynthetic activity is responsible for almost 50% of the global primary production (Field et al. 1998). The color of lakes and seas is changing, however (Roulet and Moore 2006, Kritzberg 2017, Dutkiewicz et al. 2019). In temperate and boreal regions, for instance, many clear blue lakes have changed into turbid waters due to both increasing eutrophication and an enhanced influx of organic matter (i.e., the “greening” and “browning” of lakes; Leech et al. 2018). It is likely that phytoplankton communities will be affected by these changes in the underwater light spectrum, as they consist of a taxonomically diverse set of species that deploy different photosynthetic pigments and light‐harvesting strategies (Ting et al. 2002, Kirk 2011, Croce and van Amerongen 2014). Can we use ecological theory to predict which light‐harvesting strategies will be favored in which environments?

**Figure 1:**
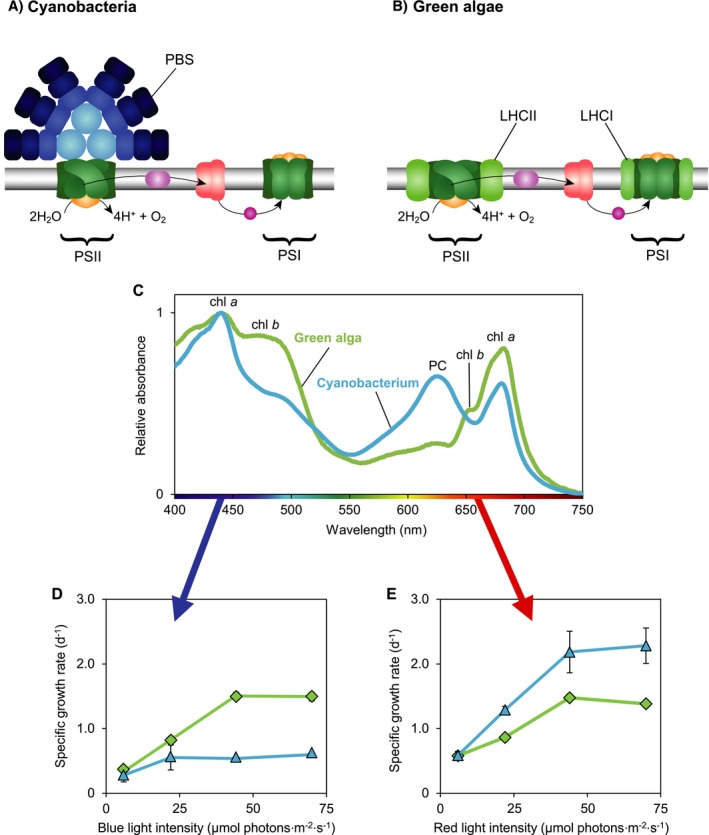
Light‐harvesting strategies of cyanobacteria and green algae. Cyanobacteria and green algae both contain two photosystems (PSI and PSII). (A) Most cyanobacteria use phycobilisomes (PBS) as light‐harvesting antennae, which transfer their absorbed light energy mostly to PSII. (B) Green algae and higher plants use chlorophyll‐based light‐harvesting complexes (LHCI and LHCII), which serve as light‐harvesting antennae for both photosystems. (C) Absorption spectra of a green alga (*Chlorella sorokiniana* 211‐8K) and a cyanobacterium (*Synechocystis* sp. PCC 6803). The spectrum of the green alga shows absorption peaks of chl *a* (440 and 678 nm) and chl *b* (480 and 650 nm), whereas the cyanobacterium shows absorption peaks of chl *a* and of the phycobili‐pigment phycocyanobilin (PC, at 625 nm). Both species also contain carotenoids, which absorb photons in the 400–500 nm range. (D) The green alga *Chlorella* (green diamonds) has higher specific growth rates in blue light, whereas (E) the cyanobacterium *Synechocystis* (blue triangles) has higher specific growth rates in red light. Data in panels D and E are averages of three biological replicates ± SD, from experiments of Luimstra et al. (2018).

All phytoplankton species use the ubiquitous pigment chlorophyll *a*, which absorbs both blue and red light (Kirk 2011). In addition, most cyanobacteria and red algae possess large light‐harvesting antennae known as phycobilisomes (PBS) (Fig. 1A). PBS contain phycobili‐pigments absorbing cyan, green, or orange‐red light, and transfer the absorbed light energy to the photosystems (Tandeau de Marsac 2003, Six et al. 2007, Watanabe and Ikeuchi 2013). Cyanobacteria allocate most of their chlorophyll *a* to photosystem I (PSI), whereas PBS are mostly coupled to photosystem II (PSII) (e.g., Myers et al. 1980, Luimstra et al. 2018). It has been hypothesized that PBS can dynamically move back and forth between PSII and PSI by state transitions (Mullineaux et al. 1997, van Thor et al. 1998), but recent research does not fully support this hypothesis (Chukhutsina et al. 2015, Ranjbar Choubeh et al. 2018, Calzadilla et al. 2019). By contrast, the cyanobacterium *Prochlorococcus*, green algae, and many other eukaryotic phytoplankton (e.g., diatoms, coccolithophores, and dinoflagellates) lack PBS, but contain light‐harvesting complexes consisting of chlorophylls and carotenoids that can effectively transfer light energy to both photosystems (Chisholm et al. 1992, Natali and Croce 2015, Nawrocki et al. 2016; Fig. 1B). Hence, we can distinguish between PBS‐based and chlorophyll‐based light‐harvesting antennae, each with their own distinct photosynthetic pigments and light absorption spectra (Fig. 1C).

Interestingly, the two contrasting light‐harvesting strategies lead to different photosynthetic efficiencies in blue light. In essence, PBS pigments do not absorb blue light < 450 nm (Tandeau de Marsac 2003, Six et al. 2007) and therefore cannot transfer the energy of blue light to PSII. Chlorophylls and carotenoids do absorb blue light. However, PBS‐containing cyanobacteria invest most of their chlorophyll *a* in PSI. Moreover, in cyanobacteria, only carotenoids of PSI appear to be involved in light harvesting, whereas carotenoids of PSII are involved in heat dissipation (Stamatakis et al. 2014). This yields an imbalance between the two photosystems, with a shortage of excitation energy at PSII and hence a low photosynthetic efficiency of PBS‐containing cyanobacteria in blue light (Pulich and van Baalen 1974, Solhaug et al. 2014, Luimstra et al. 2018, 2019; Fig. 1D). By contrast, chlorophyll‐based light‐harvesting complexes do absorb blue light and can distribute the absorbed light energy over both photosystems, which gives green algae and *Prochlorococcus* a higher photosynthetic efficiency in blue light than PBS‐containing cyanobacteria (Nawrocki et al. 2016, Luimstra et al. 2018) (Fig. 1D). These differences in photosynthetic efficiency suggest that species with chlorophyll‐based light‐harvesting antennae will have a competitive advantage in blue light.

Resource competition theory can predict the outcome of competition for nutrients and light between photosynthetic organisms (Tilman 1977, Grover 1997, Passarge et al. 2006, Jäger and Diehl 2014, Burson et al. 2018). At first, competition models treated light as a single resource (Huisman and Weissing 1994, 1995). In this case, theory predicts that the species with lowest critical light intensity is the superior competitor for light, and will competitively displace all other species (Huisman et al. 1999, Passarge et al. 2006). In reality, however, light is not a single resource but offers a spectrum of resources. Phytoplankton species exploit this environmental variation by employing different photosynthetic pigments that absorb different colors of light. Therefore, the earlier competition models were extended by incorporation of the full spectrum of light (Stomp et al. 2004, 2007). These spectral models predict stable coexistence of species if they absorb different parts of the light spectrum, a theoretical prediction that has been verified by both laboratory competition experiments and field data (Stomp et al. 2004, 2007, Burson et al. 2019).

The observation that PBS‐containing cyanobacteria absorb blue light but use it less efficiently than other colors of light (Solhaug et al. 2014, Luimstra et al. 2018, 2019) adds a new layer of physiological complexity that has not yet been considered in previous models of competition for light. It can be interpreted as a kind of luxury consumption, in which species make resources unavailable for other species even though they do not use these resources themselves (de Mazancourt and Schwartz 2012). In principle, luxury consumption can destabilize species coexistence through preemption of limiting resources, which may lead to rapid competitive exclusion and might even promote priority effects where the species that arrives first monopolizes the available resources. These considerations undermine the key assumption of earlier spectral competition models (Stomp et al. 2004, 2007) that species utilize all absorbed photons with the same photosynthetic efficiency.

Here, we develop a new resource competition model to investigate competition for light between species with different light‐harvesting strategies. In this model, contrary to the previous spectral competition models of Stomp et al. (2004, 2007), resource acquisition (light absorption), and resource use efficiency (photosynthetic efficiency) of different light colors can be varied independently. Model parameters of a freshwater cyanobacterium and a green alga, as representatives of phytoplankton with either PBS‐based or chlorophyll‐based light‐harvesting antennae, were estimated from monoculture experiments in blue and red light. The model predictions were tested in competition experiments between the two species exposed to different combinations of blue and red light. The competition experiments started from different initial conditions to examine possible priority effects resulting from luxury consumption of blue light by the cyanobacterium. To investigate the suitability of this approach for marine ecosystems, we subsequently applied the competition model to the two most abundant marine phytoplankton taxa, *Synechococcus* and *Prochlorococcus*, which also deploy PBS‐based vs. chlorophyll‐based light‐harvesting strategies.

## The Model

Our model considers a well‐mixed vertical water column, with a depth that runs from *z* = 0 at the surface to *z* = *z*_m_ at the bottom of the water column. The water column is illuminated from above, with an incident light spectrum *I*_in_(λ), where λ is wavelength. The underwater light spectrum changes with depth as a result of selective absorption of different wavelengths by phytoplankton, water, dissolved organic matter (gilvin), and suspended particles (tripton).

Let *I*(λ*,z*) denote light intensity of wavelength λ at depth *z*. According to Lambert‐Beer’s law, light intensity decreases exponentially with depth:

(1) $$I\left(\lambda ,z\right)=I_{\text{in}}\left(\lambda \right)\exp\left(-\sum_{i=1}^{n}k_{i}\left(\lambda \right)C_{i}z-K_{\text{bg}}\left(\lambda \right)z\right)$$

where *k_i_*(λ) is the specific light absorption coefficient of species *i* as function of wavelength λ (i.e., the *absorption spectrum*) and *C_i_* is the population density of species *i*. The summation term indicates that light is absorbed by *n* phytoplankton species in total, each with its own light absorption spectrum, and *K*_bg_(λ) is the wavelength‐dependent background turbidity caused by water, gilvin, and tripton. We define *I*_out_(λ) as the light intensity of wavelength λ that reaches the bottom of the water column, that is, *I*_out_(λ) = *I*(λ,*z*_m_).

The model assumes that dynamic changes in the population densities of phytoplankton species are determined by their growth and loss rates. We consider eutrophic waters in which nutrients are available in excess. The growth rates of the species can then be described as the production rates integrated over the light spectrum and averaged over depth:

(2) $$\frac{dC_{i}}{dt}=\left(\frac{1}{z_{m}}\int_{z}\int_{\lambda }p_{i}\left(\lambda ,z\right)d\lambda dz-m_{i}\right)C_{i}\;\;$$

where *i*=1,...,*n*, *p_i_*(λ,*z*) is the specific production rate of species *i* as function of wavelength λ and depth *z*, and *m_i_* is the specific mortality rate of species *i*.

The productivity of a species depends on the number of photons it absorbs and the efficiency at which it uses these absorbed photons for growth. For simplicity, we assume that the specific production rate *p_i_*(λ, *z*) is a linear function of light intensity. This simplification applies to relatively low light intensities, where light does not yet saturate photosynthesis. That is,

(3) $$p_{i}\left(\lambda ,z\right)=\phi_{i}\left(\lambda \right)k_{i}\left(\lambda \right)I\left(\lambda ,z\right)$$

where the product *k_i_*(λ)*I*(λ*,z*) describes the number of photons of wavelength λ at depth *z* absorbed by species *i*, and ϕ*_i_*(λ) is the efficiency at which absorbed photons of wavelength λ are converted into biomass production (i.e., the *photosynthetic efficiency*). The product ϕ*_i_*(λ)*k_i_*(λ) of the photosynthetic efficiency and absorption spectrum of a species is known as the *action spectrum* of that species. Cyanobacteria and eukaryotic phytoplankton all absorb both blue and red light. In contrast to most eukaryotic phytoplankton, however, PBS‐containing cyanobacteria have a much lower photosynthetic efficiency in blue than in red light.

Inserting Eq. 1 and Eq. 3 into Eq. 2, and solving the depth integral, yields

(4) $$\frac{dC_{i}}{\mathrm{dt}}=\left(\int_{400}^{700}\phi_{i}\left(\lambda \right)k_{i}\left(\lambda \right)I_{\text{avg}}\left(\lambda \right)d\lambda -m_{i}\right)C_{i}$$

where the depth‐averaged light intensity of wavelength λ, *I*_avg_(λ), is given by

(5) $$\begin{array}{cc} I_{\text{avg}}\left(\lambda \right) & =\frac{1}{z_{\text{m}}}\int_{0}^{z_{\text{m}}}I\left(\lambda ,z\right)dz\\ & =\frac{I_{\text{in}}\left(\lambda \right)-I_{\text{out}}\left(\lambda \right)}{\ln\left(I_{\text{in}}\left(\lambda \right)\right)-\ln\left(I_{\text{out}}\left(\lambda \right)\right)} \end{array}.$$

We note that *I*_avg_(λ) is monotonically related to *I*_out_(λ), that is, turbid waters with a low depth‐averaged light intensity also have a low light transmission through the water column.

In our experiments, the underwater light field consisted of a series of distinct colors instead of the full light spectrum. In this case, Eq. 4 simplifies to

(6) $$\frac{dC_{i}}{dt}=\left(\sum_{j=1}^{k}\phi_{\mathrm{ij}}k_{\mathrm{ij}}I_{\text{avg,}j}-m_{i}\right)C_{i}$$

where the production rate is summed over colors *j* = 1,…,*k* to obtain the total production of species *i*.

## Methods

### Strains and culture conditions

The PBS‐containing cyanobacterium *Synechocystis* sp. PCC 6803, which absorbs orange‐red light with the pigment phycocyanobilin, was provided by D. Bhaya (Stanford University, Stanford, California, USA). The green alga *Chlorella sorokiniana* 211‐8K, which employs chlorophyll‐containing light‐harvesting complexes, was obtained from the SAG culture collection (Göttingen, Germany). All cultures were grown at 30°C in 1.8‐L, flat‐panel chemostats under light‐limited conditions (Huisman et al. 2002), with an optical path length of *z*_m_ = 5 cm and dilution rate of *D* = 0.015 h^−1^.

The chemostats were provided with a nutrient‐rich mineral medium (BG‐11 medium; Merck, Darmstadt, Germany) supplemented with 5 mmol/L Na_2_CO_3_ and mixed by bubbling with CO_2_‐enriched air (2% v/v) flowing at a rate of 30 L/h. CO_2_ concentration of the gas mixture was monitored with an Environmental Gas Monitor (EGM‐4; PP Systems, Amesbury, Massachusetts, USA). Light was provided by LED panels (custom made by Philips Lighting BV, Eindhoven, The Netherlands) at a total incident light intensity (*I*_in_) of 45 µmol photons·m^−2^·s^−1^.

### Experimental design

The population dynamics of *Synechocystis* and *Chlorella* were studied in 12 chemostat experiments, including four monoculture experiments and eight competition experiments. First, we ran monoculture experiments with *Synechocystis* and *Chlorella* in blue LED light (450 nm) and in red LED light (660 nm). Both LED colors had a full width at half maximum of ~ 20 nm. After the monoculture experiments had reached steady state, they served as inoculum for the competition experiments. We applied four light treatments in the competition experiments, with only blue light (100% blue), only red light (100% red), and two mixtures of blue and red LEDs (33% blue/67% red and 67% blue/33% red). For each light treatment, we ran two competition experiments, one with an initial dominance of *Chlorella* (90% *Chlorella*/10% *Synechocystis*, based on biovolume) and the other with an initial dominance of *Synechocystis* (10% *Chlorella*/90% *Synechocystis*) to test for alternative stable states.

### Sampling and measurements

Light intensities transmitted through the chemostats (*I*_out_), light absorption spectra and population densities were measured almost daily during the first two weeks and subsequently two to three times per week until the end of the experiment. Total *I*_out_ integrated over 400–700 nm was measured with a LI‐250 light meter (LI‐COR Biosciences, Lincoln, Nebraska, USA) and *I*_out_ spectra were recorded using a RAMSES ACC‐VIS spectroradiometer (TriOS, Oldenburg, Germany). Light absorption spectra of phytoplankton were measured from 400‐750 nm using an updated Aminco DW2000 photospectrometer (OLIS, Bogart, Georgia, USA), and normalized to maximum absorbance at the chlorophyll *a* peak at 440 nm after subtraction of the minimum absorbance at 750 nm.

A CASY1 TTC cell counter with 60 µm capillary (Schärfe Systems GmbH, Reutlingen, Germany) was used to count cells of the monoculture experiments and measure their biovolume. To distinguish between cyanobacteria and green alga, cells in the competition experiments were counted using an Accuri C6 flow cytometer (BD Biosciences, San Jose, California, USA) equipped with a blue laser (488 nm) and red laser (640 nm). To obtain biovolumes of the two species in competition, cell counts obtained by flow cytometry were multiplied by cell volumes measured in the monocultures. Because the species differed in size, we expressed their population densities as total biovolume per liter (mm^3^/L).

### Parameter estimation

Model parameters consisted of system parameters and species parameters (Appendix S1: Table S1). System parameters (incident light intensity *I*_in_, optical path length of the chemostat *z*_m_, and dilution rate *D*) were set experimentally. We assumed that specific mortality rates (*m_i_*) of the species were dominated by the dilution rate (i.e., *m_i_* = *D*). Background turbidities in blue and red light were estimated from light transmission through chemostats filled with mineral medium but without phytoplankton using Lambert‐Beer’s law. Light absorption coefficients of the species in blue light (*k_i_*_,blue_, at 450 nm) and red light (*k_i_*_,red_, at 660 nm) were estimated from absorption spectra of the species (Fig. 1C). Photosynthetic efficiencies of the species in blue light (ϕ*_i_*_,blue_) and red light (ϕ*_i_*_,red_) were estimated by fitting the dynamic model predictions to observed trajectories of population density and blue and red light transmitted through the chemostats during the monoculture experiments, using a least‐squares method (Huisman et al. 1999, Passarge et al. 2006).

We also used the model to investigate competition between marine *Synechococcus* and *Prochlorococcus* in blue and green light. For this purpose, system parameters were the same as in the chemostat experiments, light absorption coefficients in blue light (*k_i_*_,blue_, at 450 nm) and green light (*k_i_*_,green_, at 550 nm) were estimated from measured absorption spectra of *Synechococcus* and *Prochlorococcus*. The absorption spectrum of *Prochlorococcus* was obtained from a field sample of the deep chlorophyll maximum at station ALOHA, north of the island of Oahu, Hawaii, USA (collected by M. Stomp, L. J. Stal, and J. Huisman). The *Synechococcus* spectrum was of *Synechococcus* strain WH7803 (kindly provided by L. Garczarek). Photosynthetic efficiencies in blue and green light of *Synechococcus* and *Prochlorococcus* were assumed to be similar to those in blue and red light of *Synechocystis* and *Chlorella*, respectively (Appendix S1: Table S1).

## Results

### Model analysis

#### Competition for a single color

If species compete for a single color of light, the competitive dynamics predicted by the model are straightforward. For each species *i* and color *j*, we can calculate the depth‐averaged critical light intensity $I_{avg,ij}^{*}$ at which this species remains stationary (i.e., at which *dC_i_*/*dt* = 0). According to Eq. 6, this gives

(7) $$I_{\text{avg,}ij}^{*}=\frac{m_{i}}{\phi_{\mathrm{ij}}k_{\mathrm{ij}}}$$

Similar to previous derivations (Huisman and Weissing 1994, Weissing and Huisman 1994), it can be shown that the species with lowest depth‐averaged critical light intensity is the superior competitor for this color and will competitively displace all other species.

#### Competition for two colors of light

**Figure 2:**
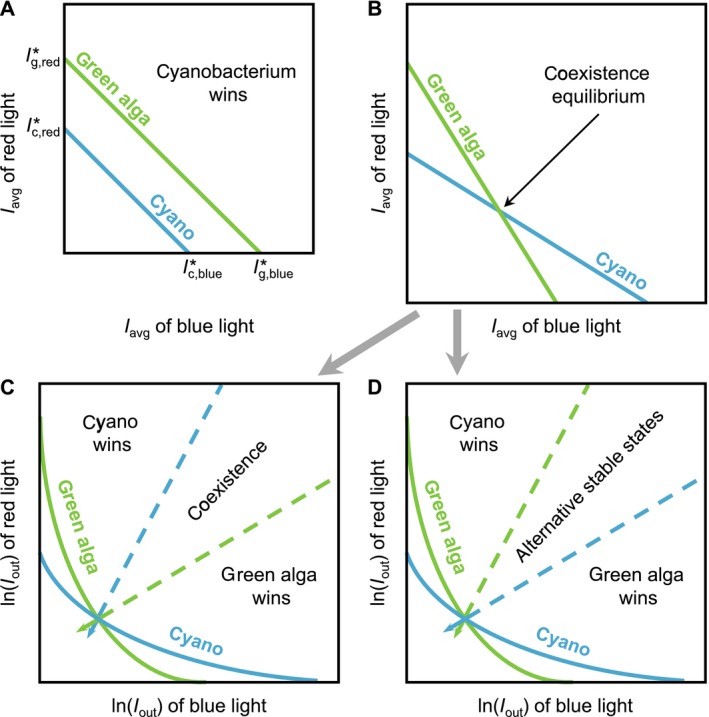
Graphical representation of competition for blue and red light between a green alga (green lines) and a cyanobacterium (blue lines). (A) The cyanobacterium is a stronger competitor for both blue and red light than the green alga (i.e., the critical light intensities $I_{\text{blue}}^{*}$ and $I_{\text{red}}^{*}$ are both lower for the cyanobacterium). In this case, the zero isoclines (solid lines) of the species do not intersect and the cyanobacterium always wins. (B) The cyanobacterium is a stronger competitor for red light, whereas the green alga is a stronger competitor for blue light. Intersection of the two zero isoclines indicates a coexistence equilibrium, which may be either stable or unstable. In panels C and D, the coordinates are transformed to light transmission *I*_out_ on a logarithmic scale. Coexistence is feasible for all supply points located in the conical region bounded by the two absorption vectors (dashed lines). (C) The coexistence equilibrium is stable if the cyanobacterium has a steeper absorption vector than the green alga. (D) The coexistence equilibrium is unstable if the green alga has a steeper absorption vector than the cyanobacterium; in this case, the system displays alternative stable states where the winner depends on the initial abundances of the species.

Now consider competition between two species, a cyanobacterium and a green alga, competing for blue and red light. The outcome of competition can be represented graphically, in style with previous resource competition models (Tilman 1982). For this purpose, we draw a resource plane with (the depth average of) blue light along the *x*‐axis and red light along the *y*‐axis (Fig. 2A, B). The zero isocline of a species is obtained by solving the equilibrium condition *dC_i_/dt* = 0 for two colors of light. According to Eq. 6, the zero isocline of the green alga is as follows:

(8a) $$I_{\text{avg,red}}=\frac{m_{g}}{\phi_{\text{g,red}}k_{\text{g,red}}}-\frac{\phi_{\text{g,blue}}k_{\text{g,blue}}}{\phi_{\text{g,red}}k_{\text{g,red}}}I_{\text{avg,blue}}$$

and that of the cyanobacterium is

(8b) $$I_{\text{avg,red}}=\frac{m_{c}}{\phi_{\text{c,red}}k_{\text{c,red}}}-\frac{\phi_{\text{c,blue}}k_{\text{c,blue}}}{\phi_{\text{c,red}}k_{\text{c,red}}}I_{\text{avg,blue}}$$

where subscripts g and c refer to the green alga and cyanobacterium and subscripts “red” and “blue” refer to red and blue light. The zero isoclines of the two species can be plotted as lines in the resource plane. The slope of the zero isocline of a species corresponds to the ratio ϕ*_i_*_,blue_ *k_i_*_,blue_/ϕ*_i_*_,red_ *k_i_*_,red_. The intercepts of the zero isocline with the *x*‐axis and *y*‐axis correspond to its critical light intensities for blue and red light, respectively (the *I* ^*^ values in Fig. 2A).

Suppose that the cyanobacterium is a better competitor (i.e., has lower critical light intensities) for both blue and red light than the green alga. In this case, the zero isocline of the cyanobacterium is located completely below the zero isocline of the green alga and the cyanobacterium will win the competition irrespective of the prevailing light color (Fig. 2A).

Now consider a scenario in which the green alga is a better competitor for blue light, whereas the cyanobacterium is a better competitor for red light. The zero isoclines are therefore arranged as in Fig. 2B, where the slope of the zero isocline of the green alga is steeper downward than that of the cyanobacterium and the two zero isoclines intersect. The intersection point represents a coexistence equilibrium (Fig. 2B). The cyanobacterium and green alga will coexist if this coexistence equilibrium is both feasible and stable.

#### Feasibility of the coexistence equilibrium

The coexistence equilibrium is feasible if the green alga and cyanobacterium can both sustain a positive population at equilibrium (i.e., if both *C*_g_ > 0 and *C*_c_ > 0). According to Lambert‐Beer’s law, in Eq. 1, light absorption is a linear function of the population densities of the species if light intensities are expressed on a logarithmic scale:

(9a) $$\ln\left(I_{\text{out,red}}\right)=\ln\left(I_{\text{in,red}}\right)-K_{\text{bg,red}}z_{m}-k_{\text{g,red}}z_{m}C_{g}-k_{\text{c,red}}z_{m}C_{c}$$

(9b) $$\ln\left(I_{\text{out,blue}}\right)=\ln\left(I_{\text{in,blue}}\right)-K_{\text{bg,blue}}z_{m}-k_{\text{g,blue}}z_{m}C_{g}-k_{\text{c,blue}}z_{m}C_{c}.$$

After some algebra, it follows from Eqs. 9a,b that the coexistence equilibrium is feasible if

(10) $$\frac{k_{\text{c,red}}}{k_{\text{c,blue}}}>\frac{\ln\left(I_{\text{in,red}}\right)-K_{\text{bg,red}}z_{m}-\ln\left(I_{\text{out,red}}^{*}\right)}{\ln\left(I_{\text{in,blue}}\right)-K_{\text{bg,blue}}z_{m}-\ln\left(I_{\text{out,blue}}^{*}\right)}>\frac{k_{\text{g,red}}}{k_{\text{g,blue}}}$$

or if both inequality signs in this equation are reversed. Here, the superscript * indicates that *I*_out,red_ and *I*_out,blue_ are evaluated at the coexistence equilibrium.

The condition in Eq. 10 can be visualized graphically if the coordinates of the resource plane are transformed logarithmically, from (*I*_avg,blue_,*I*_avg,red_) to (ln(*I*_out,blue_),ln(*I*_out,red_)) (Fig. 2C, D). We note that order is preserved when the coordinates are transformed, since *I*_avg,_ *_j_* is a monotonic function of ln(*I*_out,_ *_j_*). Hence, if zero isoclines intersect in the linear resource plane, then they intersect in the logarithmic resource plane as well. Analogous to Tilman’s (1982) consumption vectors, we can draw absorption vectors in this resource plane to describe the relative amounts of blue and red light absorbed by a species (Fig. 2C, D). According to Eqs. 9a,b, the absorption vector of the green alga has a slope *k*_g,red_/*k*_g,blue_ and that of the cyanobacterium has a slope *k*_c,red_/*k*_c,blue_. Furthermore, we can plot a supply point in the resource plane, with coordinates ln(*I*_out,blue_) = ln(*I*_in,blue_) − *K*_bg,blue_ *z*_m_ and ln(*I*_out,red_) = ln(*I*_in,red_) − *K*_bg,red_ *z*_m_. The supply point describes transmission of blue and red light through the water column prior to phytoplankton growth.

In Fig. 2C, D we have drawn two dashed lines spreading out from the coexistence equilibrium, with slopes given by the absorption vectors of the green alga (*k_g,red_*/*k_g,blue_*) and cyanobacterium (*k*_c,red_/*k*_c,blue_), respectively. According to Eq. 10, the coexistence equilibrium is feasible for all supply points that fall in the conical region bounded by these two dashed lines. The cyanobacterium (the best competitor for red light) wins for all supply points above this region, whereas the green alga (the best competitor for blue light) wins for all supply points below this region (Fig. 2C, D).

#### Stability of the coexistence equilibrium

Whether species will coexist for supply points within the conical region of Fig. 2C, D depends on the stability of the coexistence equilibrium. Previous models assumed that the photosynthetic efficiency of a species is independent of wavelength (Stomp et al. 2004, 2007). In this case, the coexistence equilibrium is always stable (Appendix S1: Section 1).

If photosynthetic efficiency varies with wavelength, stability of the coexistence equilibrium is not guaranteed. Recall that we assume that chlorophyll‐based green algae are better competitors for blue light and PBS‐based cyanobacteria for red light. In this case, the coexistence equilibrium is locally stable if the cyanobacterium absorbs relatively more red than blue light in comparison with the green alga (Appendix S1: Eq. S12):

(11) $$\frac{k_{\text{c,red}}}{k_{\text{c,blue}}}>\frac{k_{\text{g,red}}}{k_{\text{g,blue}}}$$

whereas the coexistence equilibrium is unstable if this inequality is reversed.

Graphically, this implies that stability depends on the slopes of the absorption vectors of the cyanobacterium (*k*_c,red_/*k*_c,blue_) and green alga (*k*_g,red_/*k*_g,blue_). If the absorption vector of the cyanobacterium is steeper than that of the green alga, the coexistence equilibrium is stable. In this case, the model predicts that the cyanobacterium and green algae will stably coexist (Fig. 2C). Conversely, if the absorption vector of the cyanobacterium is less steep than that of the green alga, the coexistence equilibrium is unstable. In this case, the model predicts two alternative stable states, where either the green alga or the cyanobacterium wins depending on the initial abundances of the species (Fig. 2D).

### Monoculture experiments

**Figure 3:**
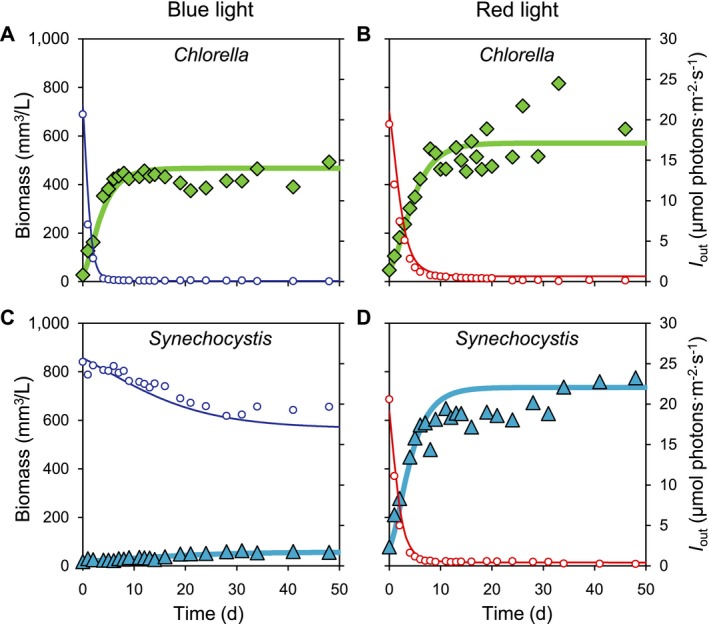
Monoculture experiments. The monocultures are of the green alga *Chlorella* (green diamonds) in (A) blue light and (B) red light, and of the cyanobacterium *Synechocystis* (blue triangles) in (C) blue light and (D) red light. Small open circles represent light transmission (*I*_out_) of blue and red light, respectively, through the monocultures. Solid lines represent model fits. Parameter values of the model are listed in Appendix S1: Table S1.

In blue light, the monoculture of the green alga *Chlorella* increased its biomass and reached steady state after ~9 d (Fig. 3A). The green alga absorbed all incident blue light, such that light transmission *I*_out_ through the monoculture was depleted to < 0.5 μmol photons·m^−2^·s^−1^ (Fig. 3A). In red light, the *Chlorella* monoculture reached a slightly higher biomass at steady state, and also depleted light transmission *I*_out_ through the monoculture to < 0.5 μmol photons·m^−2^·s^−1^ (Fig. 3B).

In blue light, the monoculture of the PBS‐containing cyanobacterium *Synechocystis* increased slowly and approached steady‐state only toward the end of the experiment (after 30 d) (Fig. 3C). At steady state, the biomass of *Synechocystis* was almost 10 times lower than that of the green alga *Chlorella* in blue light, and light transmission *I*_out_ though the monoculture remained high at ~20 μmol photons·m^−2^·s^−1^ (Fig. 3C). By contrast, in red light, the cyanobacterium *Synechocystis* increased to steady state in 10 d, produced a higher biomass in monoculture than the green alga *Chlorella*, and depleted light transmission *I*_out_ to < 0.5 μmol photons·m^−2^·s^−1^ (Fig. 3D).

Comparison of the data and model results shows that the model generally fitted well to the monoculture experiments. Parameter estimates obtained from the monoculture experiments (Appendix S1: Table S1) were used to predict the dynamics and outcome of the competition experiments.

According to our model, the species with the lowest critical light intensity for a particular color is predicted to be the best competitor for that color. The depth‐averaged critical light intensities were calculated from Eq. 7, using the parameter estimates obtained from the monocultures. In blue light, the critical light intensity was much lower for *Chlorella* than for *Synechocystis* (7.0 vs. 28.8 μmol photons·m^−2^·s^−1^; see Appendix S1: Table S2). Conversely, in red light, the critical light intensity was lower for *Synechocystis* than for *Chlorella* (9.5 vs. 10.4 μmol photons·m^−2^·s^−1^; see Appendix S1: Table S2). *Chlorella* is therefore predicted to win the competition in blue light, whereas *Synechocystis* is predicted to win in red light.

### Competition experiments

**Figure 4:**
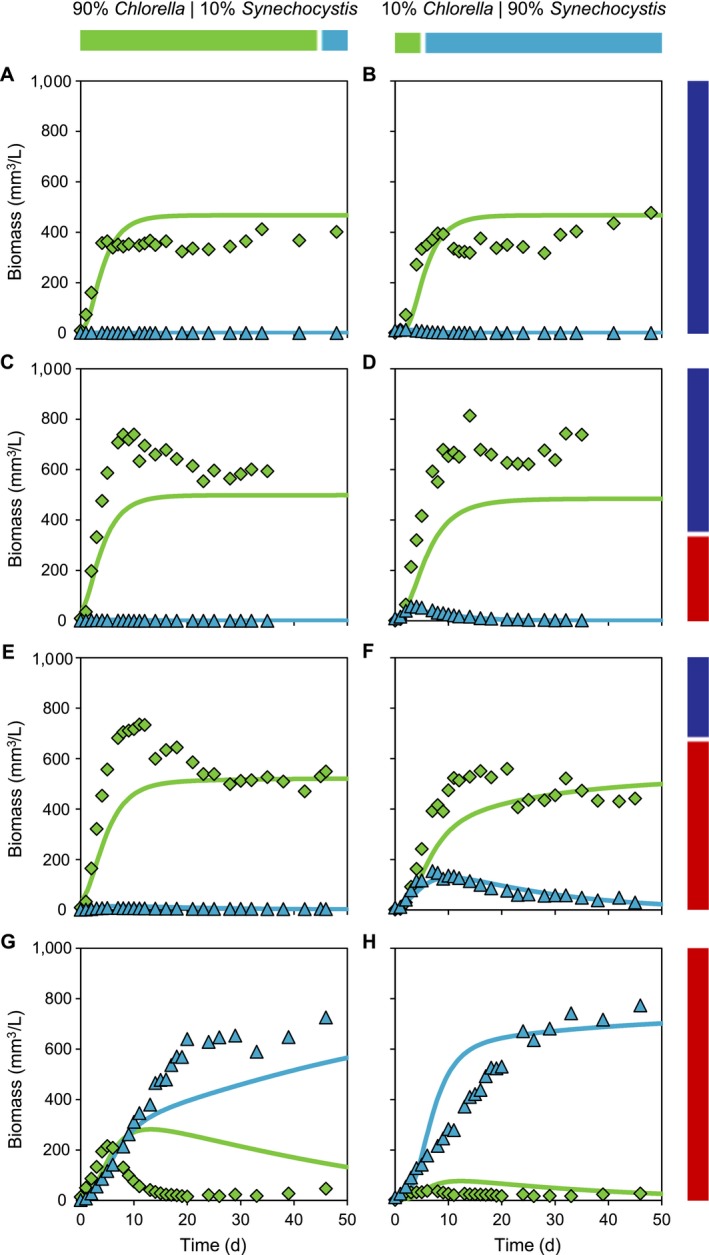
Competition experiments between the cyanobacterium *Synechocystis* (blue triangles) and the green alga *Chlorella* (green diamonds). The experiments were exposed to four different mixtures of red and blue light, as indicated by bars at the right‐hand side, with the same total light intensity. (A, B) 100% blue light, (C, D) 67% blue light and 33% red light, (E, F) 33% blue light and 67% red light, and (G, H) 100% red light. The experiments were inoculated with two different initial relative abundances of the species, as indicated by bars at the top. Panels A, C, E, and G were inoculated with 90% *Chlorella* and 10% *Synechocystis* (based on biovolume); panels B, D, F, and H were inoculated with 10% *Chlorella* and 90% *Synechocystis*. Solid lines represent model predictions based on parameters estimated from the monoculture experiments (see Appendix S1: Table S1 for parameter values). Light transmission (*I*_out_) data are presented in Appendix S1: Fig. S1.

Competition between the green alga *Chlorella* and cyanobacterium *Synechocystis* was studied at four combinations of blue and red light (Fig. 4). For each combination, we ran two experiments that differed in initial relative abundances of the two species. In blue light, *Chlorella* competitively displaced *Synechocystis* regardless of their initial relative abundances (Fig. 4A, B).

*Chlorella* was also the final winner in the experiments with 67% blue and 33% red light (Fig. 4C, D) and 33% blue and 67% red light (Fig. 4E, F). Interestingly, when *Synechocystis* represented 90% of the initial biomass, *Synechocystis* increased in biomass during the first week before it was competitively excluded by *Chlorella* (Fig. 4D, F). Furthermore, *Synechocystis* persisted longer in the experiments with 33% blue and 67% red light than in the experiments with 67% blue and 33% red light (compare Fig. 4D and Fig. 4F).

In red light, *Synechocystis* was the final winner regardless of the initial conditions (Fig. 4G, H)*.* However, when *Chlorella* dominated the initial biomass, it increased substantially during the first week before it was competitively excluded by *Synechocystis* (Fig. 4G).

The model predictions correctly predicted the winner of competition in all experiments, and also captured the competitive dynamics (Fig. 4A–H) and light transmission (Appendix S1: Fig. S1) quite well. Only in the red light experiment with 90% *Chlorella*/10% *Synechocystis*, competitive exclusion of *Chlorella* clearly occurred faster than predicted by the model (Fig. 4G). In all competition experiments, the incident light was fully absorbed by the cultures, that is, light transmission *I*_out_ was depleted to <1 μmol photons·m^−2^·s^−1^ for both blue and red light (Appendix S1: Fig. S1).

**Figure 5:**
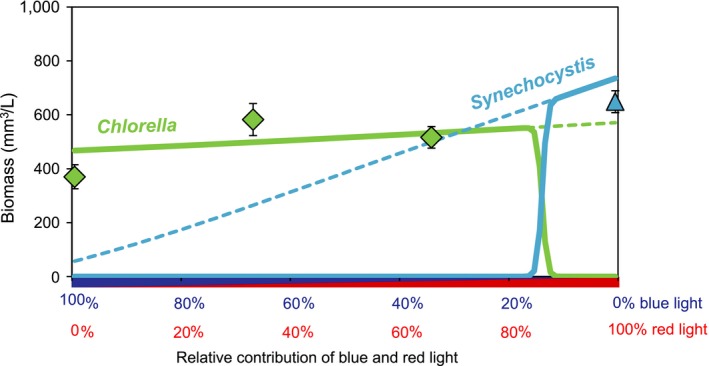
Steady‐state outcomes of the competition experiments. Solid lines show the outcome of the competition experiments between the cyanobacterium *Synechocystis* (blue line) and the green alga *Chlorella* (green line) predicted by the model, as function of the relative contribution of red and blue light. Dashed lines indicate the steady‐state biomass predicted in monoculture. Symbols show the observed steady‐state biomass of *Synechocystis* (blue triangle) and *Chlorella* (green diamonds) in the competition experiments. This steady‐state biomass was calculated as the average biomass ± SD of the last six data points in each pair of experiments (i.e., *n* = 12) in Fig. 4. Parameter values of the model are listed in Appendix S1: Table S1.

Summarizing, we find a color‐dependent reversal in species dominance (Fig. 5). In line with the experimental data, the model predicts dominance of the green alga when blue light represents a substantial portion of the underwater light spectrum, a narrow region of species coexistence, and dominance of the cyanobacterium if red light prevails while blue light constitutes less than 10% of the underwater light spectrum (Fig. 5).

### Application to marine *Prochlorococcus* and *Synechococcus*

**Figure 6:**
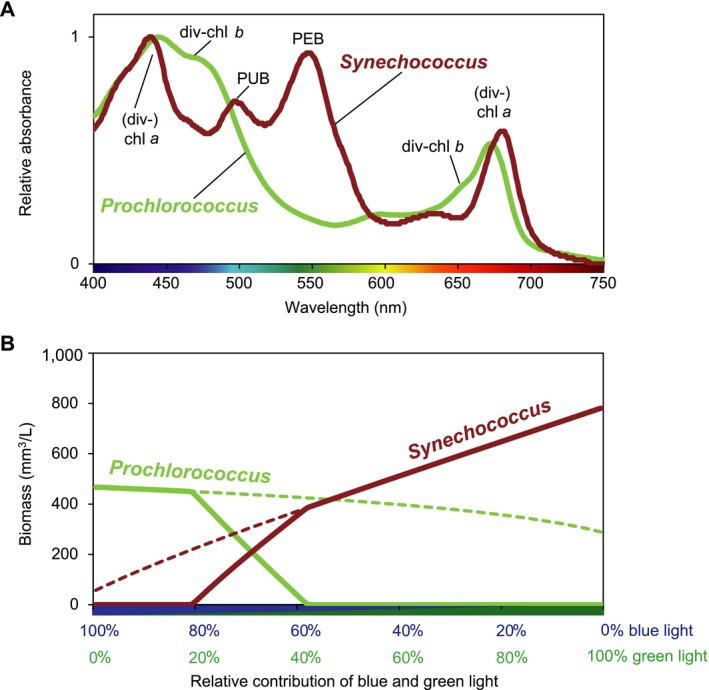
Competition between the marine cyanobacteria *Prochlorococcus* and *Synechococcus*. (A) Absorption spectra of *Prochlorococcus* and *Synechococcus*. The spectrum of *Prochlorococcus* shows absorption peaks of divinyl‐chl *a* (440 and 675 nm) and divinyl‐chl *b* (470 and 650 nm), whereas *Synechococcus* shows absorption peaks of chl *a*, phycourobilin (PUB, at 495 nm) and phycoerythrobilin (PEB, at 545 nm). (B) Model predictions of competition between *Synechococcus* (red line) and *Prochlorococcus* (green line), as function of the relative contribution of blue (450 nm) and green (550 nm) light. Solid lines show the outcome of competition; dashed lines indicate the steady‐state biomass in monoculture. The spectrum of *Prochlorococcus* was obtained from a field sample of the deep chlorophyll maximum at station ALOHA, north of the island of Oahu, Hawaii, USA, collected by M. Stomp, L. J. Stal, and J. Huisman. The spectrum of *Synechococcus* WH7803 was kindly provided by L. Garczarek.

Our resource competition model can be used to predict competition for light between the marine cyanobacteria *Synechococcus* and *Prochlorococcus* (Fig. 6). Blue and green wavelengths are the dominant light colors in most marine waters. *Prochlorococcus* is the numerically most abundant phytoplankton genus on our planet, and the ubiquitous *Synechococcus* is likely to rank second (Flombaum et al. 2013). Similar to freshwater *Synechocystis*, marine *Synechococcus* use PBS and, hence, have a low photosynthetic efficiency in blue light ≤450 nm (Luimstra et al. 2018). However, instead of the pigment phycocyanobilin, marine *Synechococcus* mostly use phycourobilin (PUB) and phycoerythrobilin (PEB), which absorb cyan (495 nm) and green light (545 nm), respectively (Six et al. 2007; Fig. 6A). *Prochlorococcus* lacks PBS but uses light‐harvesting antennae composed of divinyl‐chl *a* and divinyl‐chl *b*, which absorb blue and red wavelengths quite similar to green algae (Chisholm et al. 1992, Ting et al. 2002; Fig. 6A).

We assessed competitive interactions between *Prochlorococcus* and *Synechococcus* using mixtures of blue (450 nm) and green (550 nm) light. The model predicts that *Prochlorococcus* wins in blue light whereas *Synechococcus* wins in green light, with an intermediate region of stable species coexistence when both blue and green light are available (Fig. 6B).

## Discussion

### Comparison of theoretical and experimental results

Our results show that light color has a major effect on the outcome of competition between phytoplankton species. According to the monoculture experiments, the PBS‐containing cyanobacterium *Synechocystis* sp. PCC 6803 had a lower critical light intensity in red light, whereas the green alga *Chlorella* deploying a chlorophyll‐based light‐harvesting strategy had a lower critical light intensity in blue light. Hence, the zero isoclines of the two species will intersect (as in Fig. 2B–D). The green alga is therefore predicted to be a superior competitor for blue light, whereas the PBS‐containing cyanobacterium is predicted to be a superior competitor for red light. This prediction is confirmed by our competition experiments, where *Chlorella* won in 100% blue light (Fig. 4A, B) and *Synechocystis* won in 100% red light (Fig. 4G, H).

The model predicts that, if the zero isoclines intersect, competition for mixtures of red and blue light may lead to either stable coexistence (Fig. 2C) or alternative stable states (Fig. 2D) depending on the absorption coefficients of the species. In our study, the better competitor for red light (*Synechocystis*) absorbed relatively more red than blue light (i.e., it had a high *k*_red_/*k*_blue_ ratio; see Appendix S1: Table S1) than the better competitor for blue light (*Chlorella*). Hence, the stability criterion in Eq. is met and the model predicts stable coexistence rather than alternative stable states. To verify this prediction, each competition experiment was performed with two different initial conditions (90% *Chlorella*/10% *Synechocystis* or 10% *Chlorella*/90% *Synechocystis*). In each set of experiments, the final winner was the same regardless of the initial abundances of the species (Fig. 4). Hence, in agreement with the model predictions, luxury consumption of blue light by the PBS‐containing cyanobacterium did not lead to alternative stable states in our experiments.

Yet, stable coexistence of the two species was not observed in any of our competition experiments (Fig. 4). Why not? Previous competition studies showed that species with different photosynthetic pigments can coexist in white light (Stomp et al. 2004, 2007, Burson et al. 2019). One might therefore expect ample opportunities for species coexistence when cyanobacteria and green algae compete for different colors of light. However, our model predictions indicate that coexistence between *Chlorella* and *Synechocystis* is possible only in a very narrow range of blue and red light (Fig. 5). The underlying reason is that *Chlorella* and *Synechocystis* absorb both blue light of 450 nm and red light of 660 nm to a similar extent (Fig. 1C; Appendix S1: Table S1). As a consequence, the slopes of their absorption vectors are very similar (*k*_red_/*k*_blue_ = 0.51 and 0.55, respectively) and, therefore, the parameter region with stable coexistence of *Chlorella* and *Synechocystis* is very narrow (Fig. 5).

The model predicts a broader range of coexistence if the absorption coefficients of the competing species are more divergent. This is nicely illustrated by the model predictions for marine *Prochlorococcus* and *Synechococcus* competing for blue and green light (Fig. 6). According to their absorption spectra, *Synechococcus* absorbs more strongly in the green and hence has a much steeper absorption vector (*k*_green_/*k*_blue_ = 0.96) than *Prochlorococcus* (*k*_green_/*k*_blue_ = 0.20) (Appendix S1: Table S1). This divergence in absorption properties enables stable coexistence of the two species in a relatively broad intermediate region where both blue and green light are available (Fig. 6B).

### Underlying physiological traits

The experiments showed that the PBS‐containing cyanobacterium *Synechocystis* was the superior competitor in red light. According to the model, the competitive ability of a species for a specific light color depends on the product of its photosynthetic efficiency and light absorption coefficient for that color (see Eq. 7). In this case, the green alga *Chlorella* had a slightly higher absorption coefficient in red light than *Synechocystis*, yet the cyanobacterium *Synechocystis* was a stronger competitor because it used the absorbed red light with a distinctly higher photosynthetic efficiency (Appendix S1: Table S1). The difference in photosynthetic efficiency might be size related, as *Synechocystis* is markedly smaller than *Chlorella* (Appendix S1: Table S2). Small cells are often considered to be strong competitors for nutrients and light (Grover 1989, Litchman et al. 2010, Marañón 2015, Burson et al. 2018). For instance, small cells are less affected by the “package effect,” that is, the self‐shading of photosynthetic pigments packaged within a cell (e.g., Kirk 2011), which results in a higher light use efficiency for smaller cells (Finkel 2001, Fujiki and Taguchi 2002, Key et al. 2010, Schwaderer et al. 2011). The higher photosynthetic efficiency of *Synechocystis* might also be related to structural differences in cellular organization between prokaryotic cyanobacteria and eukaryotic algae. In cyanobacteria, the thylakoid membrane, in which the photosynthetic pigments are embedded, stretches out along the cell membrane and hence can readily absorb photons transmitted through the cell membrane (Nierzwicki‐Bauer et al. 1983, Rast et al. 2015). By contrast, in eukaryotic algae the thylakoid membrane is contained in separate organelles, the chloroplasts. The eukaryotic cell wall and extra membrane layers of the chloroplast may provide additional light‐attenuating barriers before photons reach the photosynthetic pigments (Schwaderer et al. 2011). Furthermore, eukaryotic algae will have additional costs to produce and maintain their chloroplasts and other organelles, while cyanobacteria tend to have lower maintenance requirements (Van Liere and Mur 1979).

Conversely, the green alga *Chlorella* was the superior competitor in blue light. Our model results show that this reversal in competitive dominance is a consequence of the much lower photosynthetic efficiency in blue light of *Synechocystis* (Appendix S1: Table S1). If, in our model, we assumed that *Synechocystis* had the same high photosynthetic efficiency in blue light as in red light, then application of Eq. 7 shows that the critical light intensities would have been lower for *Synechocystis* than for *Chlorella* in both blue and red light. In graphical terms, the zero isocline of *Synechocystis* would be located below the zero isocline of the green alga (Fig. 2A), and hence the cyanobacterium *Synechocystis* would have won the competition from *Chlorella* in both light colors. That was not the case, however, because PBS‐containing cyanobacteria use blue light very inefficiently. This is commonly attributed to the low absorbance of blue light ≤450 nm by the PBS, which leads to an excitation imbalance between the photosystems PSI and PSII (Solhaug et al. 2014, Luimstra et al. 2018, 2019; Fig. 1).

### Implications for natural phytoplankton communities

Our model predicts that phytoplankton species that use chlorophyll‐based, light‐harvesting antennae (e.g., green algae, diatoms, the cyanobacterium *Prochlorococcus*) are superior competitors in blue waters. Conversely, phytoplankton species that use PBS as light‐harvesting antennae (many cyanobacteria, and also red algae and glaucophytes) will be favored in green and orange‐red light environments. To what extent can these new insights aid in understanding and predicting the taxonomic composition of natural phytoplankton communities?

In recent years, dissolved organic carbon (DOC) concentrations have increased in temperate, boreal, and arctic lakes across the northern hemisphere, which has been attributed to changes in land use, acid deposition, and climate change (Monteith et al. 2007, Larsen et al. 2011, Weyhenmeyer et al. 2016, Kritzberg 2017). Light absorption by DOC decays exponentially with wavelength, with strong absorption in the blue and minor absorption in the red part of the spectrum (e.g., Kirk 2011). As a consequence, increasing DOC concentrations shift the underwater light color toward longer wavelengths, a phenomenon known as “browning” or “brownification” of lakes (Roulet and Moore 2006). Lake browning may cause major changes in phytoplankton community structure that are consistent with our model predictions. For instance, a recent study of 1,000+ lakes sampled across the continental USA showed a substantial decrease in the number of blue lakes and an increase in the number of murky lakes, caused by the simultaneous eutrophication (“greening”) and browning of lake waters (Leech et al. 2018). In agreement with our model predictions, cyanobacterial abundance was lowest in blue lakes and highest in green and murky lakes. Other studies have also shown that lake browning may result in the loss of diatoms and green algae (Urrutia‐Cordero et al. 2017), while it tends to favor cyanobacteria (Ekvall et al. 2013, Lebret et al. 2018, Feuchtmayr et al. 2019) and cryptophytes (Deininger et al. 2017, Urrutia‐Cordero et al. 2017, Wilken et al. 2018). These patterns are supported by recent competition experiments that, similar to our results, revealed that the common bloom‐forming cyanobacterium *Microcystis aeruginosa* lost the competition from a green alga in blue light, but won in orange‐red light (Tan et al. 2019). Hence, while eutrophication and global warming are often considered to be the main drivers of the increased intensity, frequency and duration of cyanobacterial blooms across the globe (O’Neil et al. 2012, Huisman et al. 2018), lake browning may play a key role as well.

The proliferation of cryptophytes in waters with high DOC concentrations is usually attributed to their mixotrophic feeding behavior (Urrutia‐Cordero et al. 2017, Wilken et al. 2018). Interestingly, however, their pigmentation is also well suited for photosynthesis in waters with high DOC concentrations. Similar to PBS‐containing cyanobacteria, cryptophytes also contain a diverse set of phycobili‐pigments (Cunningham et al. 2018, Greenwold et al. 2019), although their phycobili‐pigments are not organized into PBS‐like structures but are contained in the thylakoid lumen (van der Weij‐de Wit et al. 2006). Hence, similar to PBS‐containing cyanobacteria, cryptophytes can effectively exploit the green and orange‐red part of the light spectrum generated by relatively high DOC concentrations.

Our findings are also consistent with the biogeographical distributions of the marine cyanobacteria *Prochlorococcus* and *Synechococcus*. As predicted, these two genera coexist in many regions across the global ocean but their distributions do not completely overlap (Ting et al. 2002, Flombaum et al. 2013). In agreement with the model predictions, *Prochlorococcus* uses chlorophyll‐based light‐harvesting antennae and is particularly abundant in blue waters of the oligotrophic subtropical gyres (Partensky et al. 1999, Biller et al. 2015), whereas the PBS‐containing *Synechococcus* prevails in slightly more turbid oceanic and coastal waters dominated by greenish blue and green light (Scanlan and West 2002, Ting et al. 2002, Grébert et al. 2018). Moreover, the highly diverse *Synechococcus* genus comprises several pigment types, that have branched out over a wide range of aquatic ecosystems in accordance with the underwater light colors that can be captured by their phycobili‐pigments (Stomp et al. 2007, Grébert et al. 2018). Hence, the predominance of *Prochlorococcus* in blue ocean waters and marine *Synechococcus* in more greenish waters, with a broad intermediate region where both genera coexist, can be readily explained by their contrasting light‐harvesting strategies.

### Implementation in resource competition theory and trait‐based approaches

This work shows how the study of photosynthetic pigments and their role in light harvesting can be integrated into resource competition models and may contribute to the advancement of trait‐based approaches in freshwater and marine ecology. Trait‐based approaches provide a unifying framework to study biodiversity and community dynamics on the basis of the functional traits of species (McGill et al. 2006, Litchman and Klausmeier 2008). However, trait‐based studies in phytoplankton ecology have mostly focused on traits related to cell size and nutrient uptake (Merico et al. 2009, Kruk et al. 2010, Edwards et al. 2011). As a consequence, even though photosynthetic properties are long recognized as important traits of phytoplankton (e.g., Engelmann 1883, Edwards et al. 2015), the role of the underwater light spectrum as a major selective factor in phytoplankton communities has received surprisingly little attention (but see Stomp et al. 2004, 2007). Our results show that changes in the underwater light color of lakes and oceans may have a major and predictable impact on the photosynthetic pigments favored by natural selection and hence on the species composition of phytoplankton communities. These findings may offer a powerful trait‐based approach to study the biogeographical distribution of some of the most abundant photosynthetic organisms on our planet.
