# Supplementary material for: Changes in water color shift competition between phytoplankton species with contrasting light‐harvesting strategies
Source: Ecology. 2020 Feb 3;101(3):e02951. doi: 10.1002/ecy.2951 (PMC7079016; doi:10.1002/ecy.2951)
Supplement: Supplementary file 1 [file ECY-101-e02951-s001.pdf]

**Supporting Information.** Luimstra, V. M., J. M. H. Verspagen, T. Xu, J. M. Schuurmans, and J. Huisman. 2020. Changes in water color shift competition between phytoplankton species with contrasting light-harvesting strategies. *Ecology.*

**Appendix S1**

## Section 1: Stability analysis

### The model

We consider two species competing for two colors of light, blue (*b*) and red (*r*). In this case, the competition model of Eq. 6 can be written as:

$$\frac{dC_1}{dt} = \left(f_{1b}\left(I_{out,b}\right) + f_{1r}\left(I_{out,r}\right) - m_1\right)C_1$$
$$\frac{dC_2}{dt} = \left(f_{2b}\left(I_{out,b}\right) + f_{2r}\left(I_{out,r}\right) - m_2\right)C_2 \qquad \text{(S1)}$$

where the growth rates of species *i* on blue and red light are given by

$$f_{ib}(I_{out,b}) = \phi_{ib}k_{ib}\left(\frac{I_{in,b} - I_{out,b}}{\ln\left(I_{in,b}\right) - \ln\left(I_{out,b}\right)}\right)$$

and (S2)

$$f_{ir}(I_{out,r}) = \phi_{ir}k_{ir}\left(\frac{I_{in,r} - I_{out,r}}{\ln\left(I_{in,r}\right) - \ln\left(I_{out,r}\right)}\right)$$

### Stability analysis

The local stability of the coexistence equilibrium is investigated by analyzing the Jacobian matrix of the system (e.g., Edelstein-Keshet 1988, Otto and Day 2007). The Jacobian matrix is given by

$$\boldsymbol{J} = \begin{pmatrix} A_{11} & A_{12} \\ A_{21} & A_{22} \end{pmatrix} = \begin{pmatrix} \frac{\partial(dC_1/dt)^*}{\partial C_1} & \frac{\partial(dC_1/dt)^*}{\partial C_2} \\ \frac{\partial(dC_2/dt)^*}{\partial C_1} & \frac{\partial(dC_2/dt)^*}{\partial C_2} \end{pmatrix} \qquad \text{(S3)}$$

where the superscript * indicates that the matrix is to be evaluated at the coexistence equilibrium. The coexistence equilibrium is stable if and only if:

$$trace(\boldsymbol{J}) = A_{11} + A_{22} < 0 \qquad \text{(S4)}$$

$$det(\boldsymbol{J}) = A_{11}A_{22} - A_{12}A_{21} > 0$$

We note that, at the coexistence equilibrium, $f_{ib}(I_{out,b}) + f_{ir}(I_{out,r}) - m_i = 0$ for both species. Hence, the elements of the Jacobian matrix can be written as

$$A_{ij} = \frac{\partial f_{ib}}{\partial I_{out,b}} \frac{\partial I_{out,b}}{\partial C_j} + \frac{\partial f_{ir}}{\partial I_{out,r}} \frac{\partial I_{out,r}}{\partial C_j} \tag{S5}$$

It is straightforward to derive that both $\partial f_{ib}/\partial I_{out,b} > 0$ and $\partial f_{ir}/\partial I_{out,r} > 0$, whereas both $\partial I_{out,b}/\partial C_j < 0$ and $\partial I_{out,r}/\partial C_j < 0$. It follows that all $A_{ij} < 0$ and therefore $trace(\boldsymbol{J}) < 0$.

After some algebra, the determinant of the Jacobian matrix can be written as

$$det(\boldsymbol{J}) = \left(\frac{\partial f_{1b}}{\partial I_{out,b}} \frac{\partial f_{2r}}{\partial I_{out,r}} - \frac{\partial f_{2b}}{\partial I_{out,b}} \frac{\partial f_{1r}}{\partial I_{out,r}}\right) \left(\frac{\partial I_{out,b}}{\partial C_1} \frac{\partial I_{out,r}}{\partial C_2} - \frac{\partial I_{out,b}}{\partial C_2} \frac{\partial I_{out,r}}{\partial C_1}\right) \tag{S6}$$

From Lambert-Beer's law, we note that

$$\frac{\partial I_{out,b}}{\partial C_i} = -k_{ib} I_{out,b} \text{ and } \frac{\partial I_{out,r}}{\partial C_i} = -k_{ir} I_{out,r} \tag{S7}$$

Furthermore,

$$\frac{\partial f_{ib}}{\partial I_{out,b}} = \phi_{ib} k_{ib} \frac{\partial I_{avg,b}}{\partial I_{out,b}} \text{ and } \frac{\partial f_{ir}}{\partial I_{out,r}} = \phi_{ir} k_{ir} \frac{\partial I_{avg,r}}{\partial I_{out,r}} \tag{S8}$$

Hence, we obtain

$$det(\boldsymbol{J}) = (\phi_{1b}\phi_{2r}k_{1b}k_{2r} - \phi_{2b}\phi_{1r}k_{2b}k_{1r})\,(k_{1b}k_{2r} - k_{2b}k_{1r}) \frac{\partial I_{avg,b}}{\partial I_{out,b}} \frac{\partial I_{avg,r}}{\partial I_{out,r}} I_{out,b} I_{out,r} \tag{S9}$$

Since $\partial I_{avg,b}/\partial I_{out,b} > 0$ and $\partial I_{avg,r}/\partial I_{out,r} > 0$, the signs of the two bracketed terms in this equation determine whether the coexistence equilibrium is stable or unstable.

*Case 1: Photosynthetic efficiency independent of light color*

Suppose that photosynthetic efficiency is independent of light color, i.e., a species utilizes all its absorbed photons with the same efficiency irrespective of wavelength, as assumed by Stomp et al. (2004, 2007). Hence, $\phi_{1b} = \phi_{1r} = \phi_1$ and $\phi_{2b} = \phi_{2r} = \phi_2$, and the determinant simplifies to

$$det(\boldsymbol{J}) = \phi_1\phi_2(k_{1b}k_{2r} - k_{2b}k_{1r})^2 \frac{\partial I_{avg,1}}{\partial I_{out,1}} \frac{\partial I_{avg,2}}{\partial I_{out,2}} I_{out,1} I_{out,2} \tag{S10}$$

In this case, it follows that $det(\boldsymbol{J})>0$ and therefore the coexistence equilibrium is locally stable whenever it exists.

*Case 2: Photosynthetic efficiency depends on light color*
More generally, photosynthetic efficiency varies with light color. Let us arbitrarily assume that species 1 is a better competitor for blue light and species 2 a better competitor for red light. Graphically, this implies that species 1 has a steeper zero isocline than species 2 (as in Fig. 2B, where the green alga would be species 1 and the cyanobacterium species 2). According to Eqs. 8a,b, this difference in slope of the zero isoclines implies

$$\frac{\phi_{1b}k_{1b}}{\phi_{1r}k_{1r}} > \frac{\phi_{2b}k_{2b}}{\phi_{2r}k_{2r}} \qquad \text{(S11)}$$

Hence, the first bracketed term in Eq. S9 is positive, and therefore the sign of $det(\boldsymbol{J})$ depends only on the second bracketed term. This implies that $det(\boldsymbol{J})>0$ and, hence, the coexistence equilibrium is locally stable if

$$\frac{k_{2r}}{k_{2b}} > \frac{k_{1r}}{k_{1b}} \qquad \text{(S12)}$$

whereas it is locally unstable if this inequality is reversed. In other words, if species 2 (the better competitor for red light) absorbs relatively more red than blue light in comparison to species 1, then coexistence of the two species is stable. Conversely, if species 2 absorbs relatively more blue than red light in comparison to species 1, then the coexistence equilibrium is unstable and the winner will depend on the initial abundances of the species.

**Literature Cited**


Edelstein-Keshet, L. 1988. Mathematical models in biology. Random House, New York.

Otto, S. P., and T. Day. 2007. A biologist's guide to mathematical modeling in ecology and evolution. Princeton University Press, NJ, USA.

Stomp, M., J. Huisman, F. De Jongh, A. J. Veraart, D. Gerla, M. Rijkeboer, B. W. Ibelings, U. I. A. Wollenzien, and L. J. Stal. 2004. Adaptive divergence in pigment composition promotes phytoplankton biodiversity. Nature 432:104-107.

Stomp, M., J. Huisman, L. Vörös, F. R. Pick, M. Laamanen, T. Haverkamp, and L. J. Stal. 2007. Colourful coexistence of red and green picocyanobacteria in lakes and seas. Ecology Letters 10:290-298.

**Table S1.** Model parameters estimated from the monoculture experiments

| Symbol | Definition | Value | | Units |
|---|---|---|---|---|
| **Variables:** | | | | |
| $C_i$ | Biomass of phytoplankton species *i* | - | | $mm^3\ L^{-1}$ |
| $I_{out,blue}$ | Blue light transmitted through the chemostats | - | | $\mu mol$ photons $m^{-2}\ s^{-1}$ |
| $I_{out,red}$ | Red light transmitted through the chemostats | - | | $\mu mol$ photons $m^{-2}\ s^{-1}$ |
| **System parameters:** | | | | |
| $I_{in,j}$ | Incident blue or red light intensity | 45 | | $\mu mol$ photons $m^{-2}\ s^{-1}$ |
| $K_{bg,blue}$ | Background turbidity of blue light | 7.5 | | $m^{-1}$ |
| $K_{bg,red}$ | Background turbidity of red light | 9 | | $m^{-1}$ |
| $K_{bg,green}$ | Background turbidity of green light | 8 | | $m^{-1}$ |
| $z_{max}$ | Maximum depth of water column | 0.05 | | m |
| *D* | Dilution rate* | 0.015 | | $h^{-1}$ |
| **Species parameters[1]:** | | *Chlorella* | *Synechocystis* | |
| $\phi_{i,blue}$ | Photosynthetic efficiency in blue light | $2.30\times10^{-3}$ | $0.69\times10^{-3}$ | $mm^3\ \mu mol^{-1}$ |
| $\phi_{i,red}$ | Photosynthetic efficiency in red light | $3.00\times10^{-3}$ | $3.80\times10^{-3}$ | $mm^3\ \mu mol^{-1}$ |
| $k_{i,blue}$ | Specific light absorption coefficient in blue light | $2.60\times10^{-4}$ | $2.10\times10^{-4}$ | $m^2\ mm^{-3}$ |
| $k_{i,red}$ | Specific light absorption coefficient in red light | $1.33\times10^{-4}$ | $1.16\times10^{-4}$ | $m^2\ mm^{-3}$ |
| | | *Prochlorococcus* | *Synechococcus* | |
| $\phi_{i,blue}$ | Photosynthetic efficiency in blue light | $2.30\times10^{-3}$ | $0.69\times10^{-3}$ | $mm^3\ \mu mol^{-1}$ |
| $\phi_{i,green}$ | Photosynthetic efficiency in green light | $3.30\times10^{-3}$ | $3.80\times10^{-3}$ | $mm^3\ \mu mol^{-1}$ |
| $k_{i,blue}$ | Specific light absorption coefficient in blue light | $2.60\times10^{-4}$ | $2.10\times10^{-4}$ | $m^2\ mm^{-3}$ |
| $k_{i,green}$ | Specific light absorption coefficient in green light | $5.20\times10^{-5}$ | $2.02\times10^{-4}$ | $m^2\ mm^{-3}$ |

*We assume that specific loss rates of the species are dominated by the dilution rate of the chemostat (i.e., $m_i$=*D*)

**Table S2.** Steady-state characteristics of monoculture experiments with the cyanobacterium *Synechocystis* and green alga *Chlorella* in blue and red light.

| | *Synechocystis* | | *Chlorella* | |
|---|---|---|---|---|
| | blue light | red light | blue light | red light |
| Population density (million cells $mL^{-1}$) | 7.5 ± 0.6 | 123.4 ± 12.6 | 25.4 ± 1.9 | 31.5 ± 5.5 |
| Total biovolume ($mm^3$ $L^{-1}$) | 57 ± 4 | 696 ± 66 | 427 ± 39 | 640 ± 118 |
| Cell volume (fL $cell^{-1}$) | 7.7 ± 0.3 | 5.6 ± 0.2 | 16.8± 0.7 | 19.2 ± 1.9 |
| Light transmission $I_{out}$ (µmol photons $m^{-2}$ $s^{-1}$) | 19.3 ± 0.5 | < 0.5 | < 0.5 | < 0.5 |
| Critical light intensity $I^*_{avg,ij}$ (µmol photons $m^{-2}$ $s^{-1}$) | 28.8 | 9.5 | 7.0 | 10.4 |

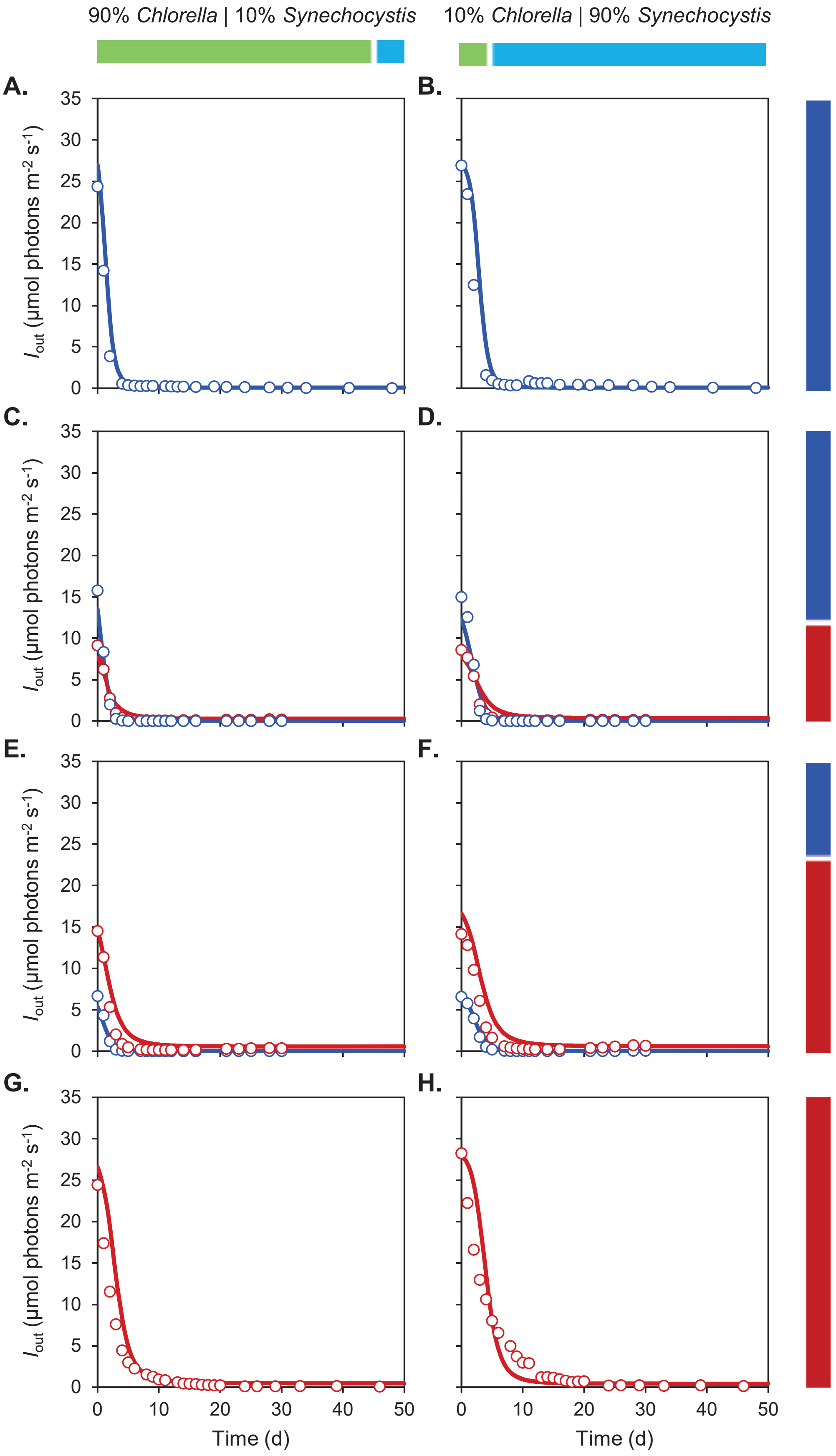


**Figure S1.** Light transmission ($I_{out}$) through the competition experiments between the cyanobacterium *Synechocystis* and the green alga *Chlorella*. Blue circles represent blue light, red circles represent red light, and solid lines represent the model predictions. The graph has the same layout as Figure 4 in the main text.
